# What is your definition of Big Data? Researchers’ understanding of the phenomenon of the decade

**DOI:** 10.1371/journal.pone.0228987

**Published:** 2020-02-25

**Authors:** Maddalena Favaretto, Eva De Clercq, Christophe Olivier Schneble, Bernice Simone Elger

**Affiliations:** Institute for Biomedical Ethics, University of Basel, Basel, Switzerland; Bielefeld University, GERMANY

## Abstract

The term Big Data is commonly used to describe a range of different concepts: from the collection and aggregation of vast amounts of data, to a plethora of advanced digital techniques designed to reveal patterns related to human behavior. In spite of its widespread use, the term is still loaded with conceptual vagueness. The aim of this study is to examine the understanding of the meaning of Big Data from the perspectives of researchers in the fields of psychology and sociology in order to examine whether researchers consider currently existing definitions to be adequate and investigate if a standard discipline centric definition is possible.

**Methods:**

Thirty-nine interviews were performed with Swiss and American researchers involved in Big Data research in relevant fields. The interviews were analyzed using thematic coding.

**Results:**

No univocal definition of Big Data was found among the respondents and many participants admitted uncertainty towards giving a definition of Big Data. A few participants described Big Data with the traditional “Vs” definition—although they could not agree on the number of Vs. However, most of the researchers preferred a more practical definition, linking it to processes such as data collection and data processing.

**Conclusion:**

The study identified an overall uncertainty or uneasiness among researchers towards the use of the term Big Data which might derive from the tendency to recognize Big Data as a shifting and evolving cultural phenomenon. Moreover, the currently enacted use of the term as a hyped-up buzzword might further aggravate the conceptual vagueness of Big Data.

## Introduction

“Big Data is like teenage sex: everyone talks about it, nobody really knows how to do it, everyone thinks everyone else is doing it, so everyone claims they are doing it …”

@Dan Ariely, 2013

Big Data is a term that has invaded our daily world. From commercial applications to research in multiple fields, Big Data holds the promise of solving some of the world’s most challenging problems. Also within academics, Big Data is popular in most disciplines, from the social sciences [1], to psychology [2], geography [3], humanities (now also called digital humanities [4]), and healthcare [5].

The possibility of using increasingly big datasets that have the potential to reveal patterns of individual and group behavior together with the promising beneficial application of data analytics [6] have attracted many researchers. Examples include the development of *smarter hospitals* where predictive analysis of Electronic Health Records (EHR) can identify in real time patients at higher risks for health deterioration or cardiac arrest [7], and the design of smarter cities projects that involve the use of aggregated data from social media, GPS, radio frequencies and consumer data to improve various sectors of urban living such as transportation, education and energy [8].

Hence, Big Data has become a frequently utilized term in the academic environment as a novel and sophisticated apparatus for research. But this raises the important question: what exactly is meant with “Big Data”?

This study aims to explore how researchers working with state of the art digital research projects in psychology and social sciences understand the term Big Data, in order to a) explore the main characteristics that researchers attribute to Big Data; b) examine whether researchers consider currently existing definitions of Big Data to be adequate; c) investigate if an overarching and straightforward discipline centric definition of Big Data in psychological and sociological research is actually possible and desirable.

The term Big Data is not a recent one. Although Diebold admits that it “probably originated in the lunch-table conversations at Silicon Graphics in the mid-1990s” [9], its first appearance in the academic literature dates back to the early 2000 in statistics and econometrics, where Big Data was used to describe “the explosion in the quantity (and sometimes, quality) of available and potentially relevant data, largely the result of recent and unprecedented advancements in data recording and storage technology” [10]. Attributed characteristics of Big Data were: *volume* (huge amounts), *velocity* (high-speed processing) and *variety* (heterogeneous data), the so-called 3Vs of Big Data [11].

In the following years, as larger quantities of data became readily available, additional definitions of Big Data were developed, that expanded on the traditional three attributes [12]: from additional Vs such as *veracity* [13], *value* [14] and *variability* [15] to other qualities including *exhaustivity* [16], *extensionality* [17], and *complexity* [18].

Despite their differences, these definitions all highlight that Big Data consists in large amounts of data coming from different sources. The European Commission defines Big Data as:

large amounts of different types of data produced from various types of sources, such as people, machines or sensors. This data includes climate information, satellite imagery, digital pictures and videos, transition records or GPS signals. Big Data may involve personal data: that is, any information relating to an individual, and can be anything from a name, a photo, an email address, bank details, posts on social networking websites, medical information, or a computer IP address [19].

Similarly, in the United States, the National Science Foundation (NSF) refers to Big Data as:

large, diverse, complex, longitudinal, and/or distributed data sets generated from instruments, sensors, Internet transactions, email, video, click streams, and/or all other digital sources available today and in the future

(NSF-12-499) [20],

or

data that challenge existing methods due to size, complexity, or rate of availability

(NSF-14-543) [21].

Despite the consensual focal point of these definitions, Big Data continues to be surrounded with conceptual vagueness due to the heterogeneous ways in which the term is used in various contexts [22]. To solve this issue, scholars have tried to propose a standard or mutually agreed upon definition of Big Data. For example De Mauro and colleagues proposed a consensual formal definition where Big Data “represents the Information assets characterized by such a High Volume, Velocity and Variety to require specific Technology and Analytical Methods for its transformation into Value” [22]. In the biomedical context, Baro et al. [23] define it exclusively by its volume and propose a threshold to over which a dataset qualifies as Big Data.

Other scholars, like Floridi for example, have criticized these traditional “attributes” definitions because they are vague and obscure and do not clarify what the term Big Data exactly means or refers to [24]. Some scholars within the social sciences have suggested to discard the “V features” definitions altogether as these attributes predominantly come from data science and data analytics and are considered too technical. Among them, one has proposed to replace them with 13 “P features” such as *portentous*, *perverse*, *personal*, *political*, *predictive*, etc. [25]. Kitchin and McArdle, argue that V-words and P-words “are often descriptive of a broad set of issues associated with Big Data, rather than characterizing the ontological traits of data themselves” [26]. The authors also claim that volume and variety are not key characteristics of Big Data—only velocity and exhaustivity are—and that the V definition is somewhat false and misleading as there are multiple forms of Big Data that do not share all the same characteristics. Moreover, it has also been argued that, as computational capacities of systems are exponentially increasing with time, it would be “impractical to define a specific threshold for Big Data volumes, because they are relative and they vary by factors, such as time and the type of data” [27], leaving the threshold to be a non-definitive and suggestive measure that is not suitable for a coherent definition.

So despite scholarly effort to narrow down the debate on the definition of Big Data and despite the existence of definitions employed by policymaking and academic bodies, such as the aforementioned definitions from the European Commission and the NSF, there is still no consensus in the literature on a proper definition of Big Data. Moreover, it is unclear to what extent academic researchers working in disciplines that embrace Big Data as a research methodology are aware of and agree with these existing definitions.

The definition of Big Data is an important topic given that Institutional Review Boards (IRBs) and regulatory bodies worldwide are struggling to regulate Big Data research and research projects involving Big Data methods and analytics. The use of growing amounts of personal data and the lack of appropriate guidelines and laws in fact raise important ethical issues [28, 29]. In psychology and sociology in particular, privacy concerns are particularly pressing. For instance the literature has highlighted the issues of linking different digital datasets that on the one hand might lead to valuable research insights but on the other reveal sensitive information about research participants [30]; some scholars have underlined the intrinsic tension between ensuring anonymity of research participants and the quality of the data set especially in light of increasingly applied policies for open data sources in academic research [31]; others have questioned the acceptability of using data from digital spaces (for instance social media) for research purposes without the subjects’ explicit consent or awareness [32]. Scandals such as Cambridge Analytica [33] and the Facebook Emotional Contagion Experiment [34] have put under the spotlight how poorly regulated research practices might jeopardize public perception of research. Public outrage that followed such scandals has led towards the development of strategies to protect both private users and research participants, both in industry and academic contexts [35]. However, researchers are still pointing to the lack of support from regulatory bodies when it comes to evaluating increasingly computational research proposals [36, 37].

As long as definitions are unclear, laws, regulations and guidelines that are bound to govern Big Data research in these two fields of research are unlikely to be effective, especially if researchers are unaware of the regulatory framework or refrain from defining their research as Big Data research out of fear for regulatory restrictions as it happened with the buzzword “nano” when referring to nanotechnology [38].

Furthermore, we should not forget that the growing datafication and digitalization of society requires researchers to work together in multidisciplinary teams in order to address the technical, ethical and legal challenges that Big Data research poses [39]. As communication challenges might arise in collective networks and among different stakeholders if each has their own definition or understanding of the discussed technology, like it happened in other scientific fields [38], the lack of a shared definition of Big Data might aggravate multidisciplinary communications. For instance if a researcher in the social sciences does not recognize that they are working with Big Data, as they have a particular definition in mind, they might be less likely to promptly and spontaneously approach expert researchers in the field of data protection and data ethics to plan improved strategies for the protection of research subjects that are in line with the standards asked by the specific privacy issues embedded in Big Data research.

For this purpose, we have conducted interviews with researchers from high standing universities both in Switzerland, and the United States. The present study offers an important contribution to the existing literature since it is one of the first studies to examine the opinions of academic researchers on the definition of Big Data in the fields of sociology and psychology.

## Methods

### Sampling

The data for this manuscript was collected as part of a larger research project on the ethics of Big Data research. The aim of the overall project was to investigate the ethical and regulatory challenges of Big data academic research in the fields of psychology and sociology in Switzerland. These two disciplines were selected not only because they are at the forefront of using Big Data methodologies in projects that involve human research subjects both directly and indirectly [40] but also because they are among the most under regulated research fields [28, 34]. This is especially true for Switzerland, the home country of the project, where Big Data research is challenging the current regulatory framework of academic research projects such as the Federal Act of Data Protection [41] and the Human Research Act [42].

We conducted 39 semi-structured interviews– 20 in Switzerland (CH) and 19 in the United States (US)–with researchers (professors, senior researchers, or postdocs) involved in research projects using Big Data methodologies in the field of psychology and sociology.

The United States were chosen as a comparative sample country where advanced Big Data research is taking place in the academic context. This instance is supported by the numerous grants that federal institutions, such as the NSF and the National Institute of Health (NIH) have been placing for Big Data research projects for several years [20, 21, 43].

Participants were selected based on their involvement in Big Data research. For this purpose, we compiled a list of keywords linked to Big Data. The list was compiled by two of the authors while performing a systematic review on Big Data that assisted the identification of the main terms related to Big Data research and technology [44]. The first author then systematically browsed the professional pages of all professors affiliated to the departments of psychology and sociology of all twelve Swiss Universities (ten Universities and two Federal Institutes of Technology) and the top ten US Universities according to the Times Higher Education University Ranking 2018 (accessed on 13.12.2018) and selected those that had these specific keywords appearing in their personal page (See Table 1):

**Table 1:** Keywords for candidate selection.

Keywords for Systematic Web Search
1. Big Data
2. Internet
3. Social Media
4. (Data) Linkage
5. Neural Networks
6. Machine Learning
7. Computational/Computer Based
8. Prediction
9. Data Mining
10. Algorithms
11. Data Analytics
12. Deep Learning
13. Profiling
14 Scoring System
15. (Algorithmic) Modelling
16. Network Analysis
17. Informatics/ Bioinformatics

For Switzerland the selection was carried out throughout January/February 2018 and for the US during January/February 2019. Other participants were identified through snowballing. Selection of the sample both through systematic selection and snowballing identified a consistent number of data scientists working on research projects involving data from human subjects in sociology, psychology and similar fields (political science, behavioral science, neuropsychology). They were therefore included in the sample as their profile matched the selection criteria. As this is not a representative sample, since it includes participants only related to the fields of psychology and sociology, we do not seek to generalize from the findings. Instead we are trying to raise awareness about the possible challenges that the use of the term Big Data is generating for research practices internationally.

A total of 194 interview invitations– 50 for Switzerland and 144 for the US—were sent via email. They contained information on the purpose of the study, participant rights, and the significance of the study. If no reply was received, a reminder was sent a week after the first invitation email. A 40% positive response rate for Switzerland and a 13.2% positive response rate for the US was obtained. We reached a sample size of 39 researchers. Regarding saturation, we define it as the point in the analysis where no new codes or themes emerge from the analysis, but only mounting instances of the same codes [45, 46]. Our interviews stopped producing new codes after analyzing the seventeenth interview of the Swiss sample and the fifteenth for the US sample, thus reaching saturation. The analysis was carried out until the end of the sample.

### Data collection

Interviews were carried out by the first and third author between January 2018 and August 2019. At the time of the interviews, the two authors were doctoral students with respectively a background in philosophy and empirical ethics and geography and computer science. Before starting the interviews, both authors were trained on interviewing skills and took formal methodological courses as part of their PhD education. Once the first pilot interviews were completed, both students received constructive feedback on their performance from two senior researchers in order to ensure the high quality of collected data.

Interviews with Swiss researchers were performed at a time and place chosen by the interviewee (usually at their home University) or via telephone, according to the participants’ preference and availability. Interviews with American researchers were carried out via Skype or telephone.

Oral informed consent was sought from all participants prior to the start of the interview and registered upon consent. From an ethical point of view, for minimal risk research involving interviews studies with experts whose data (transcripts or questionnaires) are anonymized, oral consent and active participation are ethically considered sufficient and proportionate. Furthermore, prior to the beginning of the interview phase, we asked for ethics approval to the Ethics Committee northwest/central Switzerland (EKNZ) and we received an exemption letter stating that since in Switzerland interviews with experts (not patients) are outside of the Human Research Act, they do not require ethics committee approval. To make sure that our experts were clearly informed, at the beginning of the discussion the interviewer briefly restated the purpose of the overall study, their role in the project, the confidential nature of the interview and allowed the participants to ask questions.

A semi-structured interview guide was used to conduct the interview, that was built on the experiences of the research team during prior phases of the overall project. The guide was designed through discussion and consensus within the research team after they had the time to gain familiarity with the literature and studies on Big Data research in the fields of the social sciences and psychology, and on the knowledge gained through the conduction of a systematic literature review [44].

Questions included information about (a) the research projects conducted by the interviewee either prior to or at the time of the interview, (b) the participant’s opinion on the use of social media or commercial data for academic research, (c) the researcher’s attitude towards Big Data research, (d) the participant’s personal understanding of Big Data, (e) perceived ethical, regulatory or technical barriers while conducting the research project, (f) institutional regulatory practices and experiences with Institutional Review Boards (IRBs) or Cantonal Review Boards (ECs)–the latter only for the Swiss participants, (g) the researcher’s opinion on data driven research as opposed to theory driven research. Most of the data presented in this paper comes from the questions related to topics (c) and (d), as they deal with the conceptualization, definition and understanding of Big Data. The other topics will be analyzed elsewhere. Table 2 illustrates the relevant interview questions for this article.

**Table 2:** Relevant questions from the interview guide.

Sample questions
Are you currently working on any Big Data research project?
Which one(s) of your research project(s) would you consider as involving Big Data methods or related to Big Data?
What do you think is the main difference between Big Data research and more traditional research in your field?
How would you define Big Data?

The interviews lasted between 35–90 minutes. All interviews were performed in English, being the language commonly used in academia, both for Swiss and American participants. Interviews were tape-recorded and subsequently transcribed verbatim to facilitate qualitative analysis. If participants requested, transcripts were returned to them to check the accuracy of the transcription. Only one participant asked for their transcript back and found no inconsistencies.

The transcripts were successively transferred into the qualitative analysis software MaxQDA (Version 2018) to support the analytic process [47].

### Data analysis

Applied thematic analysis was used for data analysis. This method aims at analyzing and reporting thematic elements and patterns within the data in order to organize, describe and interpret the dataset in rich detail [48]. The transcripts were therefore read in full length and independently analyzed by at least two of the members of the research group. This first step of analysis consisted of open ended coding to explore the thematic elements in the interviews. Later on the members of the team came together to confront the independent open ended coding, discuss and sort the identified themes.

Several major themes were identified from this analysis including: regulation of Big Data research, new emerging challenges, collaboration and interdisciplinary approach in digital studies, the understanding of the term Big Data, and attitudes towards Big Data studies.

Understanding and definition of Big Data were chosen to explore since the participants gave many different interpretations of the term. Subsequently, all interviews were analyzed for units of text that related both to the definition of Big Data or to expressions of attitudes or opinions towards the understanding of the term. The units were then sorted into sub-codes referring to different ways of defining or interpreting the term Big Data. This phase was carried out by the first author and checked for consistency and accuracy by the second author. Through constant discussion and comparison between the two researchers the themes were refined and systematically sorted.

## Results

For the study, a total of 39 interviews were performed including 21 sociologists (9 from CH and 12 from the US), 11 psychologists (6 from CH and 5 from the US), and 7 data scientists (5 from CH and 2 from the US). Among them, 34 were professors while 5 were postdocs or senior researchers at the time of the interview.

Of the 39 researchers, 27 explicitly stated that they were working on Big Data research projects or on projects that involve Big Data methodologies. Four participants replied that they were not involved in Big Data research and eight were unsure whether their research could be described as Big Data research (See Table 3). A significant difference was found between American and Swiss researchers: among the former, all but one confirmed their affiliation to Big Data research compared to slightly more than half (12 out of 20) of the Swiss respondents. Nevertheless, overall, no significant divergence was found between the two countries with regard to the definition of Big Data. In addition, no considerable dissimilarity was found in the answers based on the research field of the participants, with similar definitions and attitudes equally distributed over psychologists, sociologists and data scientists.

**Table 3:** Demographics.

	Sociology (S)	Psychology (P)	Data Science (D)	Total
CH Researchers	9	6	5	20
US Researchers	12	5	2	19
Professors	20	9	5	34
Postdocs/Senior researchers	1	2	2	5
Participants' self-involvement in a Big Data Project				
Yes	15	6	6	27
No	1	3	0	4
Uncertain	2	5	1	8

All, but one, participant gave an answer to the question: how would you define Big Data.

### Definitions of Big Data

First, some of our respondents initially admitted of not having a definition.

I don't think anybody really knows but I guess for me I would think that it's….

(P3US-S)

I define it as a …dataset of many features, you know, of …yeah, I don't really…It’s funny, I don't really have a definition.

(P13US-P).

A consistent minority of researchers adopted an “essential definition” of Big Data, one based on attributes or properties, while the majority of respondents supported a more “practical definition”, one that is grounded in the practices or processes related to Big Data such as data collection, data source and data processing.

Table 4 illustrates the type of definitions given by our respondents. Some overlaps occur as some participants expressed more than one key definitional trait for Big Data.

**Table 4:** Definitions.

Type of definition	Summary/Explanation	Participants
**1. Essential definition based on attributes/properties**		
1.1 Several Vs definition	Definition based on the traditional attributes of Big Data (Volume, Velocity, Variety, Veracity …)	P27CH-D; P29CH-D; P32CH-D; P33CH-S; P35CH-S.
1.2 Volume	Vast amounts of data	P39CH-S; P2US-S; P9US-S; P13US-P; P14US-P; P17US-P; P20US-S
1.3 Variety	Heterogeneous data, both structured and unstructured	P30CH-S; P34CH-D
1.4 Complexity	Very complex data compared to data that is traditionally collected in research	P5CH-S; P19US-S
1.5 Impact	Data that has a huge impact and value for society	P21US-S
**2. Practical Definitions**		
2.1 Source of Data	Data that comes from digital technologies	P25CH-P; P26CH-P; P23CH-S; P2US-S; P22US-P
2.1.1 The Human Component	Data that is generated from people	P22CH-P; P24CH-P; P37CH-S; P38CH-S P11US-P; P12US-S; P17US-P; P19US-S;
2.3 Collection	Data collected with no purpose or with no informed consent	P9CH-P; P24CH-P; P26CH-S P30CH-S; P31CH-D; P38CH-S; P3US-S; P4US-P; P5US-S;
2.4 Data Processing	Data that needs sophisticated computational processes to be analyzed	P30CH-S; P37CH-S; P2US-S; P6US-S; P16US-S; P18US-D; P19US-S; P34CH-D
2.5 Problem Solving Tool	Method that is capable of answering questions	P28CH-S; P29CH-D; P30CH-S; P31CH-D; P8US-D;

#### Essential definition based on attributes/properties

Only a few respondents referred to the traditional “several Vs” definition of Big Data: “We have big volume, we have big velocity, right? We have this kind of three V: Volume, Velocity and Variety” (P29CH-D). Some of them, used these dimensions to illustrate the many technical challenges that Big Data technologies raise.

I like the definition of the several Vs to sum it up. Big Data is simply all those data issues for which you cannot use a standard database. Right so whenever you have a problem with data and it cannot be solved with a relational database than it's a Big Data problem.

(P27CH-D)

There was no agreement among the interviewees on the number of dimensions to attribute to Big Data. One respondent acknowledged that it is uncertain how many dimensions are actually attributed to Big Data: “You know, there are always these different Vs, the 3 Vs, the 5 Vs, the 7 Vs, or whatever the 15 Rs. I don't know there's so many definitions…” (P23CH-S).

Some participants chose to describe Big Data by referring to only one of its dimensions. Of these, volume was mentioned most often, with “Big Data as being a big sample size” (P13US-P) or “Huge amounts of data usually from multiple sources” (P14US-P). Some researchers expressed the idea of a sort of undefined threshold which needs to be crossed in order for the Big Data status to be conferred: “I mean one definition is like, it's data that's too big to fit on one hard drive, or too big to be loaded on the RAM of a single machine.” (P17US-P).

However, a couple of respondents pointed out that volume or size alone are not enough to define a dataset as Big Data: “I think of Big Data studies …I realize the term focuses on the size of the dataset but I actually think of it more as the way the data are …how the data come about” (P26CH-S)

While volume was mentioned most frequently, some respondents highlighted other key characteristics such as variety or complexity:

Actually the very big part of practical work with Big Data in our context is what is sometimes referred to the variety characteristic of Big Data. So you have many sources, data comes in all kind of different formats, forms.

(P30CH-S)

Data that…complex data that you find out there compared to data that you have collected for a specific observation or experiment or so.

(P5US-S)

Finally, one participant circumscribed the definition of Big Data to its overall impact or value on research and society.

Big Data, I think to me it's more related to how big is the impact of that data. I know that is controversial. Like in research you have certain definitions that are different. I feel that's very fluid, you could have tons of data and then this data has almost no impact and the researchers do not call that Big Data.

(P21US-S)

#### Practical definitions

Most respondents, instead of focusing on the attributes ascribed to Big Data, identified some of the practical processes, such as data collection and data processes, as determinant components for the definition of Big Data.

*Source of data*. For some participants the source of data was a key factor of the definition. Some spoke for example of digital data coming from technological devices:

[…] but then my internal definition is that …it has to be …it has to draw on some kind of digital data and the analysis has to be digital in some kind of way”

(P2US-S)

Well, so Big Data are data that are generated by people when they use different technological devices.

(P25CH-P)

*The human component of Big Data sources*. A consistent number of researchers highlighted the human component and defined Big Data as data generated by people during their daily activities:

What I would probably say more classical Big Data as that when you have like a lot of … people with a lot of data points coming out of …observed situations, so …like computer behavior or like the step counts from your iPhone or the sort of that …that's more the macro perspective perhaps.

(P22CH-P)

One researcher directly referred to a specific “official” definition delivered by an academic body:

I go with the definition that is advanced here in the United States by the National Science Foundation, that Big Data is the accumulation, use, assimilation and synthesis of multi-modal, multi-leveled, multiple types of data in real-time so as to allow deep and vast analytics that are both current, retro- as well as prospective.

(P11US-P)

Within this context, some participants stated that Big Data offers traces of the real world or mirrors reality because it shows how people spontaneously behave. Others however argued that Big Data only gives a limited and sometimes incorrect representation of reality:

We try to understand the reality. And data is just one aspect of the reality, it does not reflect all reality. A typical example is that people have two phones. And so if you try to estimate the number of people travelling somewhere and you actually calculate the number of phones you need to correct for that. And if you talk to people in machine learning they just don't care about it. For their analysis the universe is the dataset. You see?

(P38CH-S)

A couple of researchers downplayed the human component by stating that Big Data is just another data structure, and not necessarily linked to the individuals producing that kind of data:

I've never done a Big Data project that I've did the data collection on. […] So by the time the data gets to me it just looks like data. So yeah, it's Big Data but it's data that I … you know, it's big in that sense and it has a lot of rows, a lot of columns …but it's you know, to me it's you know, it just looks like data. […] So yeah, for me it's just another …another data structure.

(P3US-S)

One researcher waned against understanding of Big Data as just “data” and expressed the need for critical reflection in the humanities to safeguard the people behind the dataset:

The data are also about people (…) This is really a fundamental ethical challenge to all of the social sciences and also social science history and the humanistic, digital humanities as well …the challenges for a deep rethinking, not one that refuses these new tools …but really takes on board the fact that this kind of data organizes, potentially reorganizes the entirety of the academic fields, and beyond actually. […] This is a big issue.

(P19US-S)

*Collection*. Another key feature linked to the definition of Big Data were the procedures of data collection, in particular to the absence of purpose or informed consent.

And it’s often the case with Big Data, right? You're often analyzing data that weren't originally generated for the purpose of research and now you want to use it for that purpose.

(P4US-P)

In my view Big Data is datasets which are generated from people's behavior without their informed consent.

(P9CH-P)

*Data processing*. A substantial number of respondents mentioned the typology of data analysis procedures as one of the components of the definition of Big Data. Within this view, Big Data was seen as challenging data that necessitate specific algorithmic or computational processes.

I've been defining it in sort of practical terms as data that require, you know that are in such as scale that they require some algorithmic operation on them to reduce the complexity in a format that makes it possible for you to analyze them.

(P6US-S)

I would define it data which is hard to handle. Very generally. For the practitioner.

(P30CH-S)

*Problem-solving tool*. Finally, some researchers expressed the opinion that one of the key components of the definition of Big Data is its pragmatic capacity of acting as a tool for answering questions and solving problems in a timely manner:

How easy it is to ask any question to the data that you have available. And … the more …your approach, (…) is a Big Data approach, the easier it is to answer all kinds of questions with your approach. So a good Big Data approach helps you find answers with your own data.

(P31CH-D).

Well I guess Big Data is this belief in the possibility of answering old questions or maybe new questions by just … well, by aggregating and then analyzing newly available large data sources.

(P28CH-S)

### Attitudes towards Big Data

Some of the respondents, also expressed an attitude towards the concept of Big Data either in addition to the definition or as a replacement of it.

#### The problem of conceptual confusion

Various respondents pointed to the conceptual unclarity that surrounds the term Big Data.

Especially with regards to the research environment, a couple of researchers attributed this to the various ways in which the notion is used across disciplines:

I think that every discipline would think of it differently so … in (*specific subfield of physics*) we always thought that we work with Big Data in the sense of very large datasets that need to be managed, you know, with a lot of resources. And we have a lot of complexity in that sense, right? The term though, seems to be more often applied to datasets that come from society …come from new tools and applications and instruments and society, that are just collected constantly, right? (laughs) So… it's a little bit different to the way that we were thinking about it from (*specific subfield of physics*) point of view. (…) it [the definition] depends on the context, you might refer to something different…

(P5US-S)

Due to this lack of conceptual clarity, a few researchers were reluctant to use the term Big Data: “I think it isn't a useful term because I think it confuses people (P13US-P)”.

Rather than something “useful”, various participants considered Big Data to be a popular buzzword, a cultural product of our life-world rather than a material entity:

This fuzziness is kind of interesting in itself because it kind of says something about the cultural moment we live in where everything potentially can be described, not everything, but many things can be described as Big Data, right? (…) it says some things about how present these new technologies or new ways of analyzing the world are in our daily life.

(P2US-S)

On this note, a few researchers highlighted how, especially within academics, Big Data is used to draw attention of funding agencies or research institutes:

There's also like a cynical answer about what Big Data is: whatever gets you funding.

(P17US-P)

You see it in different levels, you also see it when you have positions advertised. Because Universities and departments see it as a drawback if they don't have anyone doing kind of Big Data research. Very often new positions advertised will include that we're specifically looking for somebody who's doing this kind of research. How this research is being done …that's not something they're interested in. They just see the need to be part of the hype as it were.

(P37CH-S)

One participant believed that the conceptual confusion surrounding the term could be overcome if researchers stopped calling their work “Big Data” and started using specific subcategories (e.g. crowd sourcing, social media etc.).

I think it's important to not look at Big Data as ah "ok, you're working on Big Data". Because it's still like a huge world, that you are working on. So I understand the application is Big Data but it's nice that one goes beyond that. And like for example when talking with people who really work on crowd sourcing or social media, I think it would be really helpful when it comes to this kind of topic.

(P29CH-D)

One of the researchers, however believes that compared to the past, the meaning of Big Data is becoming clearer thanks to its increased use both by experts and laypeople. To explain what he meant the participant referred to the philosophical concept of “language games”, developed by Ludwig Wittgenstein, for whom the meaning of a word is conferred by its use within the activity of spoken and written language [49]:

So like anything else, sort of a "Wittgenstein word game", you know? … as we use the word more, the meaning of the word becomes more apparent and also evolves given the actuality of this use. So, when we started to talk about Big Data ten years ago, twelve years ago, … it was relatively amorphous and there were certain vagaries of what actually constituted a Big Data approach.

(P11US-P)

Another participant expressed this increasing understanding of what Big Data is as follows: “I think it's like pornography, you know it when you see it.” (P6US-S)

However, only one researcher expressed the belief that there is consensus among researchers in the way that the term is used and understood.

I think there's becoming more of a general consensus of an operational definition of Big Data as the term is being used more frequently. We understand what Big Data means. I mean I think there are a number sub-definitions that are possible. But I think that an overarching or undergirding definition of Big Data is probably pretty uniform at this point.

(P11US-P)

A couple of participants even asserted that Big Data is not a new concept, but that researchers have been dealing with the technical challenges of Big Data for many years:

But the concept of Big Data has been around forever. As I said it depends on your resources. You know, so when you have more information than you have resources that's Big Data. So from the very beginning we've been working on problems with Big Data.

(P8US-D)

Still, one of the researchers pointed out that, despite its longevity, Big Data is still a concept that brings novelties that need to be grasped by those working in the field:

But again it's not because they put new names on existing concepts that there is nothing new in what they do, right?

(P38CH-S)

## Discussion

Due to the regulatory and multidisciplinary challenges that Big Data is introducing in academic research, there is currently the need to explore the meaning of Big Data to facilitate the development of regulatory frameworks and that of collective research networks. This study aims to contribute to the debate on the definition of Big Data by offering a unique insight into the understanding of and attitudes towards Big Data among American and Swiss based researchers in psychology and sociology. As both Swiss and US research institutions fulfill high internationally recognized standards, we argue that their answers reflect current international discussions in this field.

The study results show that, although there was no consensus among the participants on the interpretation or definition of Big Data, some important overlaps among different definitions could be found. Taking these into consideration, there was substantial agreement among researchers in defining Big Data as huge amounts of digital data produced from technological devices that that necessitate specific algorithmic or computational processes in order to answer relevant research questions.

In spite of this agreement, researchers also reported a high amount of uncertainty and uneasiness in pinning down the term Big Data with an overarching standard definition. In the following discussion we will analyze the adequacy of the different definitions and attitudes given by our respondents in light of the literature and the issues related to ambiguities of the definition of Big Data.

Despite the fact that in the academic literature [12, 14, 22, 27, 50] and popular media [13, 18, 51] Big Data is often referred to by the several Vs definition, most of the participants in our sample did not consider this definition to be really adequate as few participants used such a definition.

In addition, even the respondents that did do so, struggled in circumscribing Big Data to a precise number of characteristics either giving a generic answer related to the “several Vs” or mentioning just one specific characteristic. This difficulty to narrow down the attributes of Big Data might come from the fact that, as the phenomenon grew in popularity, an exponentially increasing number of different features were attributed to it–“*versatility*, *volatility*, *virtuosity*, *vitality*” [52], *exhaustivity* [16], *extensionality* [17] to quote just a few—leading to confusion regarding to what are the essential characteristics of Big Data.

This may explain why most of the participants preferred a definition that was grounded in practice (e.g. data source, data collection, data processing etc.). Some of these more “practical” definitions were similar to those described in the literature. For instance, the ones that focused on data processing, showing how some of the participants associated the definition of Big Data with the purpose for which the data is used, namely Big Data analytics [53], are in line with studies that emphasize the computational needs behind the processing of large amounts of data as one of the components of the definition [12, 54]. On the other hand, responses that focused on data sources are the ones that are closer to the official definition of the European commission [19] and the National Science Foundation [20], that identify Big Data as large amounts of different type of data from different sources—emails, sensors, credit cards etc.

However, only one researcher explicitly referred to a definition of an official body, namely that of the National Science Foundation [20, 21].

The wide variety of definitions found among researchers of our sample is probably due to the fact that the term Big Data has not undergone a linear and systemic evolution but has found its meaning as a consequence of its heterogeneous utilizations in different contexts, both academic and industry related [22].

The existence of several different definitions has led to conceptual uncertainty which in turn has caused some of our respondents to reject the term altogether. This skepticism is reflected in our data as several participants admitted not having an appropriate definition for Big Data or avoided the term as much as possible—although many of them stated that they were involved in Big Data research.

This reluctance to pin down a definition or to use the term Big Data, highlights the *implicit* need to adopt a more flexible understanding of the concept of Big Data. Some researchers in fact associated Big Data with a socio-culturally evolving concept rather than with a precise fixed entity or referred to the various different disciplines in which the term is currently used. Being a culturally driven buzzword, it might not be in the nature of Big Data to have a standard definition.

Moreover, it is especially thanks to the fact that Big Data is a flexible and cluster concept that it has been able to attract researchers from various disciplines. However, due the lack of a unanimous definition, researchers might have a different understanding of Big Data, thus deteriorating the state of interdisciplinary collaboration. Although this concern was voiced by one of the participants, it was not confirmed by our research results as there were no big differences among the answers of researchers from psychology, sociology and data science with regard to the definition of Big Data. Even though the commonality of responses across the various disciplines might be attributed to the fact that most researchers were from the social sciences and other very similar disciplines, it might highlight a presumed (rather than an actual) incommensurability among disciplines.

However, as policymaking bodies are currently struggling in properly developing guidelines and regulations for Big Data [28, 29], the lack of clarity in definitions might aggravate the endeavors of IRBs worldwide as it might become difficult to strategize overarching research guidelines and regulations that could support researchers in conducting their work especially in our field of investigation namely psychology and the social sciences.

As digital technologies are becoming more and more entwined with people’s personal characteristics, their daily actions and future opportunities, Big Data research creates pressing ethical and societal issues such as privacy and data anonymity [31, 55], respect for personhood and personal identity [56], discrimination [44, 57], and informed consent [58, 59]. It is therefore of the utmost importance that scholars and regulatory bodies are aware of the harm that could be inflicted on research participants and that sustainable regulations are put in place. This might explain why the human component has become one of the main focusses of definitions of Big Data given by policymaking bodies (e.g. EU Commission 2016) [19] and academic researchers [60].

A finding that is very relevant for policy making is that many of the researchers in our sample described Big Data as personal data, or, in general, data that keeps some sort of bond with the person from whom the data was gathered. Only two researchers pointed out that they were working just with data and not with research subjects.

The acknowledgment that Big Data are personal data shows that our participants are aware of and attentive to the possible harms that could come to research if their data is not analyzed or collected properly. In fact, two researchers explicitly identified Big Data with a concern about the lack of informed consent.

Our participants’ focus on data as personal data and their awareness of the need for strategies to protect research subjects in Big Data research shows that the avoidance of the term Big Data cannot be attributed to the fear of over-regulation but seems to come exclusively from the feeling of conceptual vagueness surrounding the term. This finding is in contradiction with other studies on the definition of newly developed research technologies such as nanotechnology and biobanks which have shown that avoidance of the term is often associated with scholars fears of stricter regulations upon their research [38, 61]. In our study we found no indication of such an attitude.

Finally, a couple of researchers also highlighted that within the academic milieu Big Data is often used to attract funding from external agencies for research purposes. It is important to remember that computational social sciences [62] and digital humanities [4] were born thanks to the increased digitalization of society and that Big Data has constituted an important methodological challenge for a large number of “traditional” disciplines in the past years [52]. While we highly recognize the potential opportunities that Big Data methods are offering to multiple research fields [1–3, 6–8], the exaggerated hype for Big Data research might have also negative consequences. On the one hand, it might detract from the pressing ethical concerns that Big Data is introducing both in society and in research [55, 57, 63–65] because of the increasingly bigger promises of beneficial applications that it is offering. On the other, such hype might also aggravate the ambiguity of the term, as it is used as a catch-all to grab the attention of the listener.

In conclusion, the current flexible cultural meaning of Big Data that researchers in the fields of sociology and psychology are making use of might exacerbate the difficulty of clearly defining the term. As Kitchin and McArdle [26] interestingly note, not all Big Data share the same characteristics and there are multiple forms of Big Data—as there are of small data. This is an instance highlighted also by a couple of our respondents who argued that Big Data in its current cultural meaning it’s a tremendously vast concept that includes different subcategories and specifics that are characterized by different technical and regulatory challenges.

## Limitations

First, since our respondents were mainly from the fields of psychology and sociology, the study has overlooked the perspectives of other disciplines relevant for Big Data research, for instance medicine, nursing sciences, statistics, geography, architecture and so on. In addition, the researchers from the field of data sciences that we interviewed were strictly connected to research projects in the fields of the social sciences and psychology. Moreover, due to the interdisciplinary nature of Big Data research, it has been difficult to straightforwardly pinpoint the background of some of the researchers, as many of them have gone through a multidisciplinary academic carrier that qualifies them as experts in more than just one field of research (for instance both social sciences and data science). Finally, it must be acknowledged that the findings from this analysis are not generalizable to the understanding of Big Data of researchers in general, as they are based on only a small portion of researchers from only two disciplines. We therefore argue that more research that takes into account additional disciplines might contribute in delivering a more general picture of what is the researchers’ understanding of Big Data. However, as this is, to the best of our knowledge, one of the first studies that analyses this topic from the perspective of expert academics working in the field, we feel that it is an important contribution towards the conceptual clarification of the term Big Data.

## Conclusions

Big Data is an interdisciplinary field that requires the connection of different disciplines and the involvement of heterogeneous research skills in order to carry out projects that fully exploit the methodological novelties that Big Data is bringing to the academic environment [66]. The traditional V’s definition of Big Data was not deemed adequate by our research participants who preferred a more practical definition.

Even though most of the researchers used the term Big Data to describe their research projects, we identified an overall uncertainty or uneasiness towards the term itself. This finding might be a symptom of the tendency to recognize Big Data as a shifting and evolving cultural and scholarly phenomenon—or a cluster concept that include a plethora of sophisticated and evolving computing methodologies—rather than a clearly defined and single entity, or methodology.

We argue that assuming Big Data as a cultural evolving concept, and therefore the lack of a formal definition, does not come without issues. As Big Data is currently raising many important ethical concerns, conceptual clarity of the term Big Data would be of the outmost importance in order to strategize appropriate guidelines to protect research subjects in Big Data research in different disciplines. The use of the term Big Data as a hyped-up buzzword that is currently enacted in the academic and commercial environment might further aggravate the conceptual vagueness of Big Data.

In order to correctly capture the essence and characteristics of Big Data, it might be necessary to deconstruct or unfold the term into its different constituents, thus shifting from broad generalities to specific qualities relevant not only for scientists, but also for ethics committees and regulators. However, since to the best of our knowledge, only Kitchin and McArdle [26] have proposed this shift to a more nuanced analysis of the concept of Big Data aimed at unpacking its characteristics, we claim that more research should urgently go into this direction to gain conceptual clarity about what Big Data actually means.

## Supporting information

S1 File Interview guide. Semi structured interview guide that illustrates the main questions and themes that the researchers asked to the participants.
(PDF)

S1 Data (DOCX)

## Abbreviations

CH: Switzerland
EC: Cantonal Review Board
EHR: electronic health record
EU: European Union
HRA: Human Research Act
IRB: Institutional Review Board
NIH: National Institute of Health
NSF: National Science Foundation
US: United States
