# Supplementary material for: What is your definition of Big Data? Researchers’ understanding of the phenomenon of the decade
Source: PLoS One. 2020 Feb 25;15(2):e0228987. doi: 10.1371/journal.pone.0228987 (PMC7041862; doi:10.1371/journal.pone.0228987)
Supplement: S1 File — Semi structured interview guide that illustrates the main questions and themes that the researchers asked to the participants. (PDF) [file pone.0228987.s001.pdf]

Interview Guide for Researchers

1) Introduction:

- Can we begin with exploring your most recent research projects?/ Are you currently working on any Big Data research project?

- Which one(s) of your research project(s) would you consider as involving Big Data methods or related to Big Data?

2) Methodology and Data:

- Are you integrating data from non-academic/commercial provider (like Cumulus/Facebook/Insurance) in your project?

- Are you integrating also data from sensing devices (such as Smartphones or Fit Bit) to acquire large data sets for your research?

- One definition of big data is on the procedural level. This definition sees big Data as a set of methods for prediction. Neural networks play a key role. Have you used such algorithms? Could you elaborate on it?

<u>Anonymization</u>

- So as I understood it, you used personal data from participants/individuals during your project. How did you anonymize the samples?

3) Regulation/Guidelines and Ethics Approval:

<u>Regulation/Guidelines</u>

- Would you consider your research as Human Subject Research?

- Was it clear to you which kind of guidelines you would have to apply to your research? Are there any specific guidelines that you applied to conduct your research?

- Do you find the guidelines that you are currently using useful? Anything that bothers you about them? Do you have any suggestion on how to improve them?

<u>Ethics Approval</u>

- Did you have to ask approval to an Ethics committee to perform your research?

- Did you have to ask for approval at an institutional level (IRB)? Did you have to ask it also at a Cantonal (EC) or federal level?

- How would you describe your experience with the ethics approval process?

- Did you find your experience with this digital project in any way different from other non-digital research projects with regards to the ethics approval?

- Do you have any suggestions on how to improve the ethics approval process?

4) Societal Aspects, Ethical Considerations and Barriers:

- Have you encountered any particular challenges when conducting your research project?

    - with receiving ethics approval due to the type of data you were using?
    - With anonymization of the data?
    - Additional ethical dilemmas or challenges that came up during the various stages of the project?
    - Legal challenges? Are there any barriers created by the law?

- How do you think data research should be ideally regulated?

- What are in your opinion the minimal requirements that the law should enact to ensure that data research is carried out with minimal challenges but fulfilling ethical requirements?

<u>The concept of Hypothesis and the influence of Big Data on research</u>

- In light of the ease of obtaining data with growing technological innovations, we now have the possibility to carry out data driven studies and discard the concept of hypothesis driven research. The idea is that "the numbers talk for themselves". What is your opinion on the matter?

    - is this situation changing the way research is performed in your field?
    - do you feel that data driven research has an added value for science and society?
    - do you think such studies should be conducted differently from other more classical research?

- What do you think is the main difference between Big Data research and more conventional research in your field? Do you think this has any implications for the guidelines?

- Considering the complexity involved in accessing and managing data research from different sources, who in your opinion, owns the data?

- How would you define Big Data?
