# Supplementary material for: What is your definition of Big Data? Researchers’ understanding of the phenomenon of the decade
Source: PLoS One. 2020 Feb 25;15(2):e0228987. doi: 10.1371/journal.pone.0228987 (PMC7041862; doi:10.1371/journal.pone.0228987)
Supplement: S1 Data — (DOCX) [file pone.0228987.s002.docx]

The raw data (audio-files) and the transcripts related to the project cannot be openly released due to ethical constraints (such as easy re-identification of the participants and the sensitive nature of parts of the interviews). The data will be kept for three years after the finalization of the study (in accordance with the ethics application).

The data is stored on a safe server to which only the team members of the NRP 75 Project Elger have access to. The university servers are backed up regularly by the IT service of the University of Basel to ensure quality preservation of the data for the stipulated three years after the finalization of the study.

Although the authors cannot make their study’s data publicly available at the time of publication, all authors commit to make the data underlying the findings described in this study fully available without restriction to those who request the data, in compliance with the PLOS Data Availability policy. For data sets involving personally identifiable information or other sensitive data, data sharing is contingent on the data being handled appropriately by the data requester and in accordance with all applicable local requirements. We therefore commit to provide sections of our data which form the basis of analysis in this particular manuscript whenever requested.

Upon request, a data sharing agreement will be stipulated between the Institute for Biomedical Ethics and the one requesting the data that will state that:

1) The shared data must be deleted by the end of 2023 as stipulated in the recruitment email sent to the study participants designed in accordance to the project proposal of the NRP 75 sent to the Ethics Committee northwest/central Switzerland (EKNZ) .

2) The people requesting the data agree to ensure its confidentiality, they should not attempt to re-identify the participants and the data should not be shared with any further third stakeholder not involved in the data sharing agreement signed between the Institute for Biomedical Ethics and those requesting the data.

3) The data will be shared only after the Institute for Biomedical Ethics has received specific written consent for data sharing from the study participants.

The authors are the point of contact for fielding data access requests as they are the ones that collected the data and that have access to the safe server where the data is stored. The main points of contact for this manuscript are the Head of the Institute for Biomedical Ethics (Bernice Elger: [b.elger@unibas.ch](mailto:b.elger@unibas.ch)) and the corresponding author (Maddalena Favaretto: [maddalena.favaretto@unibas.ch](mailto:maddalena.favaretto@unibas.ch)).
